# Caring for Alaska Native prostate cancer survivors in primary care: a survey of Alaska Tribal Health System providers

**DOI:** 10.3402/ijch.v73.23637

**Published:** 2014-02-27

**Authors:** Jon C. Tilburt, Stacy Kelley, Christine A. DeCourtney, Katherine M. Humeniuk, Jerilyn Latini, Simon P. Kim

**Affiliations:** 1 Division of General Internal Medicine, Mayo Clinic, Rochester, MN, USA; 2 Biomedical Ethics Program, Mayo Clinic, Rochester, MN, USA; 3 Knowledge & Evaluation Research Unit, Mayo Clinic, Rochester, MN, USA; 4 Robert D. and Patrician E. Kern Center for the Science of Healthcare Delivery, Mayo Clinic, Rochester, MN, USA; 5 Clinical and Research Services, Alaska Native Tribal Health Consortium, Anchorage, AK, USA; 6 Department of Urology, Alaska Native Medical Center, Anchorage, AK, USA; 7 Department of Urology, Yale University, New Haven, CT, USA

**Keywords:** prostate cancer, Alaska Native, cancer survivorship

## Abstract

**Background:**

Little is known about the constraints of optimizing health care for prostate cancer survivors in Alaska primary care.

**Objective:**

To describe the experiences and attitudes of primary care providers within the Alaska Tribal Health System (ATHS) regarding the care of prostate cancer survivors.

**Design:**

In late October 2011, we emailed a 22-item electronic survey to 268 ATHS primary care providers regarding the frequency of Prostate Specific Antigen (PSA) monitoring for a hypothetical prostate cancer survivor; who should be responsible for the patient's life-long prostate cancer surveillance; who should support the patient's emotional and medical needs as a survivor; and providers’ level of comfort addressing recurrence monitoring, erectile dysfunction, urinary incontinence, androgen deprivation therapy, and emotional needs. We used simple logistic regression to examine the association between provider characteristics and their responses to the survivorship survey items.

**Results:**

Of 221 individuals who were successfully contacted, a total of 114 responded (52% response rate). Most ATHS providers indicated they would order a PSA test every 12 months (69%) and believed that, ideally, the hypothetical patient's primary care provider should be responsible for his life-long prostate cancer surveillance (60%). Most providers reported feeling either “moderately” or “very” comfortable addressing topics such as prostate cancer recurrence (59%), erectile dysfunction (64%), urinary incontinence (63%), and emotional needs (61%) with prostate cancer survivors. These results varied somewhat by provider characteristics including female sex, years in practice, and the number of prostate cancer survivors seen in their practice.

**Conclusions:**

These data suggest that most primary care providers in Alaska are poised to assume the care of prostate cancer survivors locally. However, we also found that large minorities of providers do not feel confident in their ability to manage common issues in prostate cancer survivorship, implying that continued access to specialists with more expert knowledge would be beneficial.

Prostate cancer is the most common non-skin cancer in men in the United States (1). Although the incidence of prostate cancer among Alaska Native men is lower than the US population, mortality is higher (2). While white men survive longer than other races (i.e. African–American, Hispanic, and American Indian/Alaska Native), survival rates for different races are similar when corrected for grade and stage. The stage at diagnosis predicts 5-year disease-specific survival rates: local stage disease, 100%; regional stage, 94%; and metastatic disease, 31% (3). Many patients with prostate cancer are asymptomatic at diagnosis and it is recommended that primary care providers engage in an informed discussion with their patients as **screening** does increase the number of men diagnosed with non-metastatic, **early** disease (4). Furthermore, policy makers have highlighted the importance of survivorship care, partially managing consequences of primary treatment in primary care. With survivorship growing in importance, it is imperative to know the perceived capacity of primary care providers to engage in survivorship care.

The Alaska Native Tribal Health System (ATHS) provides birth to death care for approximately 130,000 Alaska Natives/American Indians residing in the 500,000+ square mile state. It is a primarily roadless state with immense geographic barriers from the Arctic environment in the north, mountain ranges and moderate rain forests in the southern part of the state. They also provide health care for non-Natives when there are no private options. The ATHS includes physicians located at the Alaska Native Medical Center (ANMC), a tertiary care hospital, and 6 regional primary care hospitals. Rural hospital physicians make a few trips each year to the villages within the region, while ANMC physicians travel to regional hospitals to hold specialty clinics. Others including nurse practitioners and physician assistants work at ANMC, regional hospitals, and at sub-clinics located in a few of the larger villages. The primary care providers in the remote hospitals do not have the network of support city hospitals provide. They must make decisions daily on a wide range of medical issues presented to them and often have insufficient time to provide on-going support to patients.

Alaska Native men in rural Alaska who are referred for screening must travel long distances to see ANMC urology specialists for diagnosis and treatment if an abnormality is identified. Our previous outreach work with prostate cancer survivors in Alaska raised issues in care coordination for these patients. Specialists who are most skilled are overburdened and hours away. Local doctors know them well but seemed to have variable levels of confidence managing symptoms and sequelae after treatment.

Treatment for prostate cancer involves important decision-making trade-offs and potential long-term sequelae requiring on-going management. Poor patient–provider communication in prostate cancer may result in lack of informed treatment choices, may lead to care that does not elicit patient priorities, and may also result in treatments that are either too aggressive (i.e. result in functional outcome disparities) or not aggressive enough (i.e. result in survival disparities). The care of cancer survivors is an important priority identified by national bodies such as the National Cancer Institute (NCI), Institute of Medicine (IOM), and American Cancer Society (ACS) (5–7). For patients diagnosed with prostate cancer, they confront multiple health issues ranging from cancer surveillance and quality of life concerns following treatment.

Ideally, the medical care following primary therapy for prostate cancer would involve multidisciplinary medical care with urologists, radiation oncologists, medical oncologists and primary care providers. Caring for survivors in rural and geographically dispersed contexts with limited budgets as is the case in the ATHS, however, may be challenging. In these settings, primary care providers may serve as an even more important clinical link for prostate cancer survivors in particular, dealing with a variety of functional and quality of life concerns. Primary care providers are often the only link to oncologists due to geographic constrains and that specialists are only available in Anchorage and Fairbanks.

Despite NCI, IOM, and ACS priorities to optimize the care of cancer survivors, little is known about the actual constraints of doing so in Alaska and specifically in primary care settings. With limited resources and availability of specialists and as part of larger collaborations surrounding care delivery in prostate cancer, ANMC urologists requested support from the Mayo Clinic on how to best provide urology consult support services to regional primary care providers who suspected a diagnosis or prostate cancer and/or needed to provide on-going care to Alaska Native men diagnosed with prostate cancer. Ascertaining the degree of comfort and identifying potential gaps that primary care providers feel about prostate cancer survivorship may improve the quality of care. The purpose of this study was to describe the experiences and attitudes of primary care providers within the ATHS regarding the care of prostate cancer survivors.

## Methods

### Study participants & data collection

The Mayo Clinic and Alaska Area Institutional Review Boards approved this study. The research team was comprised of a general internist, 2 urologists, and cancer researchers with public health backgrounds. We compiled a list and obtained email addresses for all primary care providers (including physicians, nurse practitioners, and physician assistants) currently practicing in the ATHS. In some cases, regional hospitals’ lists of provider employees’ email addresses were not up-to-date. Reliability of email service varies among regional corporations and some providers use their personal email for work purposes.

The survey was developed in August 2011 and underwent pilot testing with a small group of providers comprised of 4 general internists, an oncologist, and a family nurse practitioner. Feedback from providers was used to reword survey questions and how to incentivize participation. In preparation for survey distribution, ANMC Medical Director/Urologists mailed out an introductory letter notifying all Tribal Health Directors that the survey would be distributed to all primary providers within their facility and to encourage their participation. In late October 2011, we emailed a link to a 22-item electronic survey to this sample of providers. Providers who completed the survey were entered in a raffle to win one of three gift certificates for a complimentary flight to any destination within the United States. Providers who did not respond to the first invitation to participate were sent up to 3 subsequent reminder messages. Providers who did not respond after the second email were also sent a paper version of the electronic survey. We also enlisted the support of administrative coordinators to help encourage provider participation at regional sites and included them in a separate raffle for a complementary flight. The process and incentive techniques used were drawn from survey research techniques aimed at reaching a busy provider population that is often inundated with survey requests from researchers interested in experiences in a remote setting.

### Survey instrument

The electronic survey instrument for this study was developed using REDCap electronic data capture tools hosted at Mayo Clinic (8). REDCap (Research Electronic Data Capture) is a secure, web-based application designed to support data capture for research studies.

Through an iterative process of literature review, question formulation, pilot testing with primary care and specialty care providers, and subsequent question revision, our research team developed a 22-item survey containing questions about ATHS providers’ views on prostate cancer survivorship. We also included questions about providers’ demographic characteristics (i.e. age, gender, practice location, specialty, years in practice, and average number of patients seen in a clinic day), as well as providers’ degree of agreement with a statement about patient adherence: “The majority of my patients follow through on provider recommendations” (“Strongly disagree,” “Somewhat disagree,” “Somewhat agree,” “Strongly agree”).

### Primary measures

Providers completed 8 questions on survivorship issues in prostate cancer. These included 5 items that asked providers to consider a clinical scenario about a married male patient who “underwent a radical prostatectomy in 2002 for intermediate risk prostate cancer at age 63 years (Stage II, Gleason 7, margin negative)” who is “now 72 with diabetes, mild obesity, and hypertension” and who, since his surgery, has “moderate erectile dysfunction and mild urinary incontinence.” Respondents were asked to select the frequency with which they would order a PSA test (“Every 6 months,” “Every 12 months,” “Every 18 months,” “Every 24 months”), who should be responsible for the patient's life-long prostate cancer surveillance (“Primary care provider,” “Internal medicine provider,” “Medical oncologist,” “Urologist” or “Other”), and who ideally should support the patient's emotional health and medical needs as a prostate cancer survivor (“Onsite specialist visits,” “Support groups for patients,” “Survivorship clinics,” “Prostate cancer survivor retreats,” or “Other”).

Respondents were also asked to rate their level of comfort addressing prostate cancer recurrence monitoring, erectile dysfunction, urinary incontinence, and emotional needs with prostate cancer survivors like the patient in the clinical scenario (“Not at all comfortable,” “A little comfortable,” “Moderately comfortable,” “Very comfortable”), their level of confidence in “managing men receiving androgen deprivation therapy for prostate cancer as long as there is a medical oncologist available” (same scale as noted above, using “confident” instead of “comfortable”), and to estimate the number of prostate cancer survivors in their regular patient load (“0,” “1–3,” “4–7,” “8 or more”).

### Analysis

Responses from those providers who completed the paper version of the survey were manually entered into the REDCap system. Survey responses were exported from REDCap into SAS version 9.2 (SAS Institute, Cary, NC), with which all basic descriptive statistics were calculated. Bivariate tests of association were performed using simple logistic regression models. For ease of analysis, 4-point response scales were subsequently dichotomized.

## Results

Of the 268 potential respondents, 47 could not be contacted due to undeliverable addresses. Of the remaining 221 participants, a total of 114 providers returned completed surveys for a response rate of 52% (114/221). Characteristics of respondents are shown in Table I. We were unable to compare characteristics of non-respondents to respondents because non-respondent demographic information was not available. Provider respondents were primarily female (64%), practice as physicians (56%), see more than 11 patients every day (74%), and have been in practice for more than 10 years (61%). A majority of respondents also report seeing between 1 and 3 prostate cancer survivors in their regular patient load (54%), and agree with the statement “the majority of my patients follow through on provider recommendations” (67%) (Table II).

**Table I:** Characteristics of 114 survey respondents as well as their patient population

Characteristic	No. (%)
Sex	
Male	41 (36)
Female	73 (64)
Provider type	
Physician	64 (56)
Family practice	58 (91)
General practice	1 (2)
Internal medicine	2 (3)
Other	3 (5)
Nurse practitioner	16 (14)
Physician assistant	33 (29)
Other	1 (1)
Years in practice	
Less than 1 year	1 (1)
1–2 years	5 (4)
3–5 years	19 (17)
6–10 years	19 (17)
More than 10 years	69 (61)
Average daily no. patients seen	
5 or less patients	9 (8)
6–10 patients	19 (17)
11–15 patients	58 (51)
16–20 patients	22 (19)
More than 20 patients	5 (4)
In your regular patient load, about how many are prostate cancer survivors?	
0	23 (22)
1–3	56 (54)
4–7	14 (14)
8 or more	10 (10)
The majority of my patients follow through on provider recommendations	
Strongly disagree	4 (4)
Somewhat disagree	31 (30)
Somewhat agree	59 (57)
Strongly agree	10 (10)

**Table II:** Attitudes and practices of ATHS providers regarding prostate cancer survivor care

Survey item & response	No. (%)			
Case study scenario*				
If you were monitoring George, how often would you order his PSA test (assuming he remained asymptomatic)?				
Every 6 months	28 (27)			
Every 12 months	72 (69)			
Every 18 months	0 (0)			
Every 24 months	4 (4)			
In the ideal situation, who should be responsible for George's life-long prostate cancer surveillance?				
Primary care provider	62 (60)			
Internal medicine provider	1 (1)			
Medical oncologist	6 (6)			
Urologist	35 (34)			
In the ideal situation, which of the following would best support George's emotional health as a prostate cancer survivor?				
Onsite specialist visits	14 (14)			
Support groups for patients	64 (63)			
Survivorship clinics	16 (16)			
Prostate cancer survivor retreats	8 (8)			
Other				
In the ideal situation, which of the following would best support George's medical needs as a prostate cancer survivor?				
Onsite specialist visits	73 (71)			
Support groups for patients	6 (6)			
Survivorship clinics	14 (14)			
Prostate cancer survivor retreats	1 (1)			
Other	9 (9)			
Issues in prostate cancer survivor care				
How confident are you in managing men receiving androgen deprivation therapy for prostate cancer as long as there is a medical oncologist available?				
Not at all confident	22 (21)			
A little confident	34 (33)			
Moderately confident	40 (38)			
Very confident	8 (8)			
Indicate your level of comfort in addressing the following topics with prostate cancer survivors:	Not at all comfortable	A little comfortable	Moderately comfortable	Very comfortable
Monitoring for prostate cancer recurrence	7 (7)	36 (35)	39 (38)	22 (21)
Erectile dysfunction	4 (4)	31 (30)	50 (49)	18 (17)
Urinary incontinence	6 (6)	32 (31)	53 (51)	12 (12)
Emotional needs	10 (10)	30 (29)	48 (46)	16 (15)

* Case study: George underwent a radical prostatectomy in 2002 for intermediate risk prostate cancer at age 63 years (Stage II, Gleason 7, margin negative). He is now 72 with diabetes, mild obesity, and hypertension. Since his surgery, he has moderate erectile dysfunction and mild urinary incontinence (1–2 pads per day). He is married. ATHS= Alaska Tribal Health System.

When considering the clinical scenario previously mentioned, most ATHS providers would order a PSA test every 12 months (69%) and believed that, ideally, the patient's primary care provider should be responsible for his life-long prostate cancer surveillance (60%), though many providers also selected a urologist as the responsible individual (34%). Support groups were selected by 63% of respondents as being the best mode of support for the patient's emotional health, while onsite specialist visits were selected as being the best mode of support for the patient's medical needs by the greatest number of providers (71%).

Regarding topics specific to prostate cancer survivorship care, slightly over half of surveyed providers indicated that they are either “not at all” or “a little” confident managing men receiving androgen deprivation therapy for prostate cancer even when there is a medical oncologist available (54%). However, a majority of providers report feeling either “moderately” or “very” comfortable addressing topics such as prostate cancer recurrence (59%), erectile dysfunction (64%), urinary incontinence (63%), and emotional needs (61%) with prostate cancer survivors.

These results varied somewhat by characteristics of respondents including sex, years in practice, and the number of prostate cancer survivors seen in their practice (Table III). Women were significantly more likely than men to report being not at all or a little comfortable monitoring for prostate cancer recurrence (OR 5.3; 95% CI 2.1–13.8) and addressing erectile dysfunction (OR 7.3; 95% CI 2.3–23.0). Compared to newer clinicians with just 0–5 years of experience, those in practice longer were significantly less likely to report being not comfortable monitoring for prostate cancer recurrence (ORs 0.1 & 0.3; 95% CIs 0.03–0.7, 0.1–0.9, for 6–10 years and more than 10 years in practice, respectively). None of the respondent characteristics included in our analyses were found to distinguish those lacking confidence in managing men receiving Androgen Deprivation Therapy (ADT), addressing urinary incontinence, or emotional needs (Table III).

**Table III:** Unadjusted tests of association

	OR (95% CI)				
	
Characteristic	Not at all or a little confident managing men receiving ADT for prostate cancer	Not at all or a little comfortable monitoring for prostate cancer recurrence	Not at all or a little comfortable addressing ED	Not at all or a little comfortable addressing urinary incontinence	Not at all or a little comfortable addressing emotional needs
Sex					
Male	1.0	1.0	1.0	1.0	1.0
Female	3.6 (1.6–8.3)	5.3* (2.1–13.8)	7.3* (2.3–23.0)	3.8 (1.5–9.9)	1.6 (0.7–3.7)
Provider type					
Physician	1.0	1.0	1.0	1.0	1.0
Non-physician	2.0 (0.9–4.4)	1.9 (0.8–4.1)	1.0 (0.4–2.3)	0.7 (0.3–1.6)	1.4 (0.6–3.1)
Years in practice					
0–5 years	1.0	1.0	1.0	1.0	1.0
6–10 years	1.9 (0.4–8.0)	0.1 (0.03–0.7)	0.4 (0.1–1.7)	0.1 (0.01–1.0)	0.7 (0.2–3.0)
More than 10 years	0.8 (0.3–2.0)	0.3 (0.1–0.9)	0.7 (0.3–1.9)	0.9 (0.3–2.5)	0.8 (0.3–2.2)
Average daily no. patients seen					
10 or less patients	1.0	1.0	1.0	1.0	1.0
11–15 patients	0.9 (0.4–2.4)	0.5 (0.2–1.4)	1.1 (0.4–3.0)	1.5 (0.6–4.2)	2.2 (0.8–6.0)
16–20 patients	0.5 (0.2–1.8)	0.9 (0.3–2.9)	0.7 (0.2–2.7)	1.3 (0.4–4.6)	2.0 (0.6–6.9)
More than 20 patients	0.7 (0.1–6.0)	1.0 (0.1–8.2)	0.6 (0.1–7.0)	1.1 (0.1–14.3)	0.9 (0.1–10.2)
In your regular patient load, about how many are prostate cancer survivors?					
0	1.0	1.0	1.0	1.0	1.0
1–3	0.9 (0.3–2.5)	0.5 (0.2–1.3)	0.6 (0.2–1.7)	0.8 (0.3–2.2)	1.2 (0.4–3.1)
4–7	0.4 (0.1–1.4)	0.04 (0.01–0.4)	0.4 (0.1–1.8)	1.0 (0.3–3.7)	0.9 (0.2–3.4)
8 or more	0.3 (0.1–1.4)	–	–	–	0.2 (0.02–1.6)
The majority of my patients follow through on provider recommendations					
Strongly/somewhat disagree	1.0	1.0	1.0	1.0	1.0
Strongly/somewhat agree	0.5 (0.2–1.1)	0.4 (0.2–1.0)	0.4 (0.2–1.0)	0.7 (0.3–1.6)	0.4 (0.2–1.0)

* p < 0.001.

## Discussion

In this survey of primary care providers in the ATHS, we found general agreement about the frequency of post-treatment PSA monitoring (i.e. every 12 months) and the preferred individual in charge of life-long prostate cancer surveillance (i.e. primary care provider) for a hypothetical prostate cancer survivor described in a clinical scenario. This finding is important since PSA monitoring is essential following primary therapy for localized prostate cancer given the lifelong risk for biochemical recurrence. Furthermore, primary care providers in a geographic area with limited access to urologists and radiation oncologists agreed with PSA monitoring intervals consistent with clinical guidelines (9). While we had no particular hypotheses regarding sex, we also found that respondents’ comfort level discussing topics relevant to prostate cancer survivorship may be associated with it. Conversely, the finding that newer providers are less comfortable speaks to accumulated experience. If a doctor has taken care of 1–2 patients, that may be important, compared to a seasoned provider. This raises the possibility of tailoring outreach preferentially to subgroups of early career providers.

Another key aspect of medical care for prostate cancer survivorship is managing the quality of life issues following primary therapy. Indeed, several studies have demonstrated high rates of treatment-related quality of life issues following surgery and radiation therapy (10, 11). In our study, we also found relatively high levels of comfort among ATHS providers in discussing topics such as erectile dysfunction, urinary incontinence, emotional needs, and PSA monitoring among prostate cancer survivors.

In our study, we also demonstrate that the 1 area of medical care where primary care providers expressed the highest degree of discomfort involved treating men with primary androgen deprivation therapy. A majority of respondents stated they had limited or no confidence in managing primary androgen deprivation therapy and related long-term sequelae, such as metabolic syndrome, coronary artery disease to osteoporosis (12–15). The use of primary androgen deprivation therapy following surgery has become increasingly controversial unless used for overt metastasis or rapidly progressing PSA. It is therefore understandable that primary care providers would express less confidence about managing prostate cancer patients with luteinizing-hormone-releasing hormone (LH-RH) agonists.

This study has several limitations. First, our relatively small sample size limits the generalizability of our findings beyond that of primary care providers in Alaska. Furthermore, our response rate was moderate, but with a fairly high number of undeliverable mailings. This could be due to logistical issues related to the maintenance of reliable email, lack of updating of provider lists or high provider turnover rates. If the survey was repeated elsewhere, the opinions reported may differ; however, we feel that this is unlikely due to the independent nature of the ATHS email system and that there were no significant differences between “new” (i.e. 1–2 years in practice) primary care providers and “experienced” (10 or more years in practice) providers and their responses to the survey. We also chose to design a survey consisting of primarily close-ended (i.e. forced-choice) questions, which may have limited our ability to more richly assess the potential gaps or barriers that primary care providers in Alaska experience or perceive in their care of prostate cancer survivors. Further research utilizing mixed methods or a purely qualitative design may help address this limitation and shed further light on the experience of Alaska primary care providers.

Overall, these data suggest that most primary care providers in Alaska are poised to assume the care of prostate cancer survivors locally but with specialty back-up. Nevertheless, large minorities of providers – some of whom are the sole provider for hundreds of miles around – do not feel confident in their ability to manage common issues in prostate cancer survivorship. Physician turnover in these positions is high and often includes a number of physicians who just completed their training. Rigorous testing of health care delivery interventions including telemedicine and Continuing Medical Education (CME) trainings that link local patients and primary care providers to specialists as well as care delivery innovations that incentivize high quality survivorship care may be sustainable and plausible ways to improve care for remote communities in a cost effective manner. Further research to test such models may be warranted.
